# A before and after comparison of the effects of forest walking on the sleep of a community-based sample of people with sleep complaints

**DOI:** 10.1186/1751-0759-5-13

**Published:** 2011-10-14

**Authors:** Emi Morita, Makoto Imai, Masako Okawa, Tomiyasu Miyaura, Soichiro Miyazaki

**Affiliations:** 1 Department of Preventive Medicine, Nagoya University Graduate School of Medicine, 65 Tsurumai-Cho, Showa-Ku, Nagoya, 466-8550, Japan; 2 Department of Psychiatry, Shiga University of Medical Science, Seta Tsukinowa-cho, Otsu, Shiga 520-2192, Japan; 3 Department of Sleep Medicine, Shiga University of Medical Science, Seta Tsukinowa-cho, Otsu, Shiga, 520-2192, Japan; 4 Faculty of Science and Technology, Ryukoku University, 1-5 Yokotani, Seta Oe-cho, Otsu, Shiga, 520-2194, Japan

**Keywords:** forest walking (Shinrin-yoku), actual sleep time, actigraphy, St. Mary's Hospital Sleep Questionnaire, circadian phase

## Abstract

**Background:**

Sleep disturbance is a major health issue in Japan. This before-after study aimed to evaluate the immediate effects of forest walking in a community-based population with sleep complaints.

**Methods:**

Participants were 71 healthy volunteers (43 men and 28 women). Two-hour forest-walking sessions were conducted on 8 different weekend days from September through December 2005. Sleep conditions were compared between the nights before and after walking in a forest by self-administered questionnaire and actigraphy data.

**Results:**

Two hours of forest walking improved sleep characteristics; impacting actual sleep time, immobile minutes, self-rated depth of sleep, and sleep quality. Mean actual sleep time estimated by actigraphy on the night after forest walking was 419.8 ± 128.7 (S.D.) minutes whereas that the night before was 365.9 ± 89.4 minutes (n = 42). Forest walking in the afternoon improved actual sleep time and immobile minutes compared with forest walking in the forenoon. Mean actual sleep times did not increase after forenoon walks (n = 26) (the night before and after forenoon walks, 380.0 ± 99.6 and 385.6 ± 101.7 minutes, respectively), whereas afternoon walks (n = 16) increased mean actual sleep times from 342.9 ± 66.2 to 475.4 ± 150.5 minutes. The trend of mean immobile minutes was similar to the abovementioned trend of mean actual sleep times.

**Conclusions:**

Forest walking improved nocturnal sleep conditions for individuals with sleep complaints, possibly as a result of exercise and emotional improvement. Furthermore, extension of sleep duration was greater after an afternoon walk compared to a forenoon walk. Further study of a forest-walking program in a randomized controlled trial is warranted to clarify its effect on people with insomnia.

## Introduction

Sleep disturbance is a major health issue in Japan. In the general population, a national survey in 1997 showed that the prevalence of insomnia, including difficulty initiating sleep, difficulty maintaining sleep, and early morning awakening, was 21.4% [1]. Sleep disturbances have been reported to be a risk factor for depression and suicide [2-7]. Therefore to obtain good mental health among the general population, it is important to improve sleep among those who have sleep complaints, rather than only among insomnia patients. Concrete and practical methods to improve sleep that are applicable in daily life are necessary. For example, habitual exercise was associated with sleep condition in the general population and exercise programs improved self-rated quality of sleep in the elderly [1,8,9].

It is known that natural environments have various beneficial effects on human health. European countries and Japan have a long tradition of health resort programs to optimize health conditions, with a natural environment recognized as relevant to such facilities [10]. A green environment has been reported to have beneficial effects on human health. Mitchell and Popham [11] reported an association between the amount of green spaces in residential areas and health status in a population study in the UK. All-cause mortality and mortality from circulatory diseases were lower in populations living in the greenest areas. Ulrich [12] reported that surgical patients assigned to rooms with windows looking out on natural scenery with trees had shorter postoperative hospital stays, received fewer negative evaluative comments in nurses' notes, and took fewer potent analgesics than those in similar rooms with windows facing a brick wall. Moreover, Kuo and Taylor [13] reported that green outdoor settings appear to reduce attention-deficit/hyperactivity disorder symptoms in children.

In Japan, forests cover 68.2% of the land area [14]. Forest walking is a common recreational activity in Japan because it is considered to promote both physical and mental health by breathing in the substances released from trees and through exercise and/or other healing factors associated with forest environments [15,16]. Indeed, according to an opinion poll conducted in Japan in 2007, 36.2% of respondents had participated in forest walking in the previous 1 year [17]. Recent studies revealed that the physiological and psychological benefits of forest walking are due mainly to stress reduction. Forest walking was shown to increase natural killer (NK) cell activity and immunoglobulin levels [16]. Blood pressure, pulse rate, and salivary cortisol concentration were lower in people in a forest compared with a city area [18]. Moreover, Multiple Mood Scale-Short Form (MMS) [19,20] scores of friendliness and wellbeing were higher, and MMS score of depression and State-Trait Anxiety Inventory-State Scale (STAI-S) [21] scores were lower on the forest-walking day compared with the control day, especially in individuals who felt chronic mental stress [22]. Another study showed that walking in a forest is more effective for decreasing blood glucose levels than other activities, such as exercise on a cycle ergometer or treadmill or underwater exercise [15]. Forest walking and presence in a natural environment may improve sleep because of their efficacy in stress reduction and the physical exercise involved. However, few studies have evaluated the effect of forest walking on sleep.

The aims of this before-after study were to evaluate the acute effects of forest walking for a community-based population with sleep complaints and to compare the effects between forenoon and afternoon forest walking, because the time of day when exercise is undertaken is one factor related to sleep response [23].

## Method

### Participants and Location

Participants were community-based adults who had self-rated sleep complaint(s). They were recruited mainly through personal communications, regional advertisements, and the Internet. Eighty-three healthy volunteers agreed to participate in this study. Twelve participants who did not complete St. Mary's Hospital Sleep Questionnaire (SMHSQ) twice and did not wear a wrist actigraph were excluded from the analysis. Therefore, suitable data were available for 71 participants (M/F, 43/28; aged in their 10 s, n = 3; 20 s, n = 7; 30 s, n = 13; 40 s, n = 6; 50 s, n = 17; 60 s, n = 17; 70 s, n = 5; no data, n = 3). The mean ± SD score of the Pittsburgh Sleep Quality Index (PSQI) was 6.4 ± 3.3 (n = 49). The score for 28 participants (57.1% of those available) was ≥6, which is above the cutoff point. Forty-one participants took part in a forenoon session whereas 30 did so in an afternoon session. Only one participant took a sleeping drug the night after forest walking. Of the participants, 49 individuals wore wrist actigraphs (see below), 42 of whom were included in the analysis (M/F, 24/17; no data, n = 1; aged in their 10 s, n = 1; 20 s, n = 5; 30 s, n = 8; 40 s, n = 4; 50 s, n = 9; 60 s, n = 10; 70 s, n = 4; no data, n = 1).

The study was carried out in the "Ryukoku Forest" of Ryukoku University in Shiga Prefecture, located in the western part of Honshu, the main island of Japan. The elevation differential over the walking areas within the forest was approximately 35 meters. The forest-walking program was conducted on eight separate weekend days from September to December 2005, and was held in two daily sessions: a forenoon session between 10:00 and 12:00 and an afternoon session between 14:00 and 16:00. Participants voluntarily took part in either of the two sessions. Each walking program lasted approximately 2 hours. We provided two types of program: a 2500-meter walking program and a 900-meter walk with some light work such as felling small trees using a hand saw within the forest program. The participants were accompanied by 2-3 guides from Ryukoku University.

We obtained informed consent from all participants. This study was approved by the ethics committee of Shiga University of Medical Science.

### Outcome Measures

The time schedule of the questionnaire survey is presented in Table 1. Sleep characteristics were compared between the nights before and after walking in the forest to evaluate the immediate effects of forest walking. All participants were required to fill in the Japanese version of SMHSQ on the night before and the night after forest walking [24-27]. The questionnaire consisted of self-rated sleep depth (1 = very light to 7 = deep), number of awakenings (0 = not at all to 7 = more than six times); sleep quality ("How well did you sleep last night?" 1 = very badly to 6 = very well); alertness on waking ("How clear-headed did you feel after getting up this morning?" 1 = very drowsy to 6 = very alert); satisfaction with sleep (1 = very unsatisfied to 5 = completely satisfied); early morning awakening (yes/no); and difficulty falling asleep (1 = none or very little to 4 = extreme difficulty). The questionnaire for the night before forest walking was collected at the forest walking session whereas that for the night after forest walking was collected by mail.

**Table 1:** Time schedule of questionnaire

	Day of forest walking		Next day
	Before	After	
SMHSQ	X		X (in the morning)
PSQI	X		
STAI-S	X	X	
Borg Scale		X	

SMHSQ: St. Mary's Hospital Sleep Questionnaire; PSQI: Pittsburgh Sleep Quality Index; STAI-S: State-Trait Anxiety Inventory-State.

Among participants in the 8 survey days, those who took part in 4 specific survey days were also requested to wear a wrist actigraph (Actiwatch-64; Mini Mitter Company, Inc, OR, USA) to estimate actual sleep time, sleep efficiency, immobile minutes, and sleep latency in addition to completing the above self-rated questionnaires [28-30]. Actigraphy measurement was carried out for a period totaling 6 days, from 3 days before to 2 days after forest walking. The epoch length of the actigraphy was set at 2 minutes. Data were analyzed by Actiware-Sleep version 5.0 (Mini Mitter).

### Other Measurements

Besides socio-demographic characteristics, all participants were required to complete the Japanese version of the Pittsburgh Sleep Quality Index (PSQI) questionnaire related to self-rated sleep quality during the past 1 month [31-34]. The cutoff point of PSQI is 5.5. Higher scores are associated with worse quality of sleep. Participants were also requested to provide information on exercise habits (1 = rarely to 5 = > 20 minutes every day). We defined habitual exercise as exercising for 20 minutes more than once a week, but without taking into account the intensity of the exercise [35,36].

STAI-S (score range, 20-80) evaluations were performed just before and immediately after 2 hours of forest walking to investigate the effects of forest walking on perceived psychological states [21,37]. Participants also completed the Borg scale to examine perceived exertion immediately after forest walking [38].

### Statistical Analysis

Participants who completed SMHSQ both the night before and the night after forest walking or who wore a wrist actigraph were included in the analysis. Although actigraphy was carried out for a total of 6 nights, the night just before and that immediately after forest walking were compared by paired-Student's t test. SMHSQ item scores from the nights before and after forest walking were also compared by paired Wilcoxon signed-rank test, or McNemar's test. In addition, STAI-S scores before and those immediately after 2 hours of forest walking were compared by paired-Student's t test.

To compare the effects on sleep between forenoon and afternoon sessions, repeated measures analysis of variance (ANOVA) was conducted using the measurement points (repeated factor: the night before forest walking vs. that after) × the forest walking session (between-subject factor: forenoon vs. afternoon). To explore other relevant factors related to the immediate measurable effects on sleep after forest walking, repeated measures analysis of variance (ANOVA) was conducted using the measurement points of outcome items observed as significant in the above analysis (repeated factor: the night before forest walking vs. that after) × a between-subject factor such as sex, age (< 60/≥60 years), exercise habits, daily disturbances of sleep (PSQI, < 5.5/≥5.5), score of STAI-S decreased by forest walking (below/above average), Borg scale after forest walking (below/above average), forest walking on Saturday vs. Sunday, and forest walking alone vs. forest walking with light work.

Additional repeated measures ANOVA were conducted. The repeated factor was one of the relevant sleep parameters identified in above analysis (sleep depth, sleep quality of SMHSQ, and actual sleep time and immobile time by actigraphy), and between-subject factors were the decrease in score of STAI-S by forest walking (below/above average) and forest walking session (forenoon vs. afternoon). SPSS 14.0J for Windows and IBM SPSS Statistics 19 were used for statistical analysis; significance level was set at 5%.

## Results

### Comparison of sleep between the nights before and after forest walking

The results of self-rated sleep conditions measured by SMHSQ comparing the night before to that after forest walking are shown in Table 2. The scores of self-rated depth of sleep and sleep quality were significantly improved the night after forest walking. The results of objective sleep analysis by actigraphy are shown in Table 2. Actual sleep time and immobile minutes the night after forest walking were significantly longer than the night before. Mean actual sleep time the night before was 365.9 ± 89.4 (S.D.) minutes whereas the night after was 419.8 ± 128.7 minutes. Mean immobile time the night before forest walking was 356.3 ± 89.1 minutes whereas the night after was 410.2 ± 127.7 minutes. Median sleep start time did not change (23:48 vs. 23:46; n = 42), whereas median sleep end time delayed approximately 20 minutes the night after forest walking compared with the night before (6:58 vs. 7:18). Among participants with actigraphy, 38.5% in the forenoon session (10 of 26) reported in their sleep diary that they had a nap(s) after forest walking whereas 28.6% of participants (4 of 14) in the afternoon session reported napping.

**Table 2:** Sleep characteristics for the night before and the night after forest walking

	*n*	Night before forest walking	Night after forest walking	Statistical test	Comment	
Actual sleep time (min)	42	365.9 ± 89.4	419.8 ± 128.7	*p *= 0.02^a^		
Actual sleep (%)	42	86.9 ± 7.4	88.0 ± 6.9	*p *= 0.4^a^		
Sleep latency (min)	42	20.1 ± 32.7	10.4 ± 9.7	*p *= 0.07^a^		
Immobile time (min	42	356.3 ± 89.1	410.2 ± 127.7	*p *= 0.02^a^		
Sleep start time (median)	42	23:48	23:46			
Sleep end time (median)	42	6:58	7:18			
St. Mary's Hospital Sleep Questionnaire						
Self-rated depth of sleep (score)	64	4.7 ± 1.4	5.1 ± 1.5	*p *= 0.03^b^	1: Very light	7: Deep
Number of awakenings (times)	66	1.48 ± 1.24	1.45 ± 1.34	*p *= 0.7^b^	0: Not at all	7: More than six times
Sleep quality (score)	63	4.0 ± 1.1	4.3 ± 1.2	*p *= 0.04^b^	1: Very badly	6: Very well
Alertness on waking (score)	62	3.4 ± 1.2	3.6 ± 1.3	*p *= 0.054^b^	1: Very drowsy	6: Very alert
Satisfaction with sleep (score)	63	3.3 ± 1.0	3.4 ± 1.1	*p *= 0.3^b^	1: Very unsatisfied	5: Completely satisfied
Early morning awakening (n, % yes)	60	10, 16.7%	8, 13.3%	*p *= 0.7^c^	1: No	2: Yes
Difficultly falling asleep (score)	64	1.3 ± 0.5	1.2 ± 0.5	*p *= 0.3^b^	1: None or very little 4: Extreme difficulty	

Mean ± SD, a: Paired-Student's t-test, b: Paired Wilcoxon signed-rank test, c: McNemar's test.

### Psychological effects of 2-hours forest walking based on changing STAI-S scores just before and immediately after forest walking

The psychological effects of forest walks were based on comparison of STAI-S given just before and immediately after a 2-hour walk. The STAI-S scores significantly decreased with 2 hours of forest walking, from 37.2 ± 9.3 to 30.2 ± 6.1 (Student's paired-t test: *p *< 0.001; n = 47). Mean Borg scale score immediately after forest walking was 11.6 ± 2.3. Given that 11 points is considered fairly light, the intensity of the forest walking in this study was considered light exercise [38].

### Comparison of effects between forenoon and afternoon participation

The sleep conditions estimated by actigraph and SMHSQ between forenoon and afternoon sessions are presented in Table 3. Regarding objective sleep parameters measured by actigraphy, the interactions between measurement points (the night before forest walking vs. the night after) and the forest walking session (forenoon vs. afternoon) on actual sleep time (*p *= 0.005) and immobile minutes (*p *= 0.006) were significant. Mean actual sleep times and mean immobile minutes did not increase after forenoon walks whereas they increased after afternoon walks.

**Table 3:** Comparison of effects on forenoon and afternoon walking

		Forenoon session			Afternoon session		Interaction^c^
	n	Before^a^	After^b^	n	Before^a^	After^b^	
Actual sleep time (min)	26	380.0 ± 99.6	385.6 ± 101.7	16	342.9 ± 66.2	475.4 ± 150.5	0.005
Actual sleep (%)	26	88.8 ± 5.1	88.9 ± 5.5	16	83.9 ± 9.6	86.6 ± 8.7	0.28
Sleep latency (min)	26	22.8 ± 40.2	11.1 ± 10.9	16	15.6 ± 14.3	9.4 ± 7.3	0.61
Immobile time (min)	26	372.2 ± 95.7	377.5 ± 98.2	16	330.6 ± 72.8	463.4 ± 153.6	0.006
St. Mary's Hospital Sleep Questionnaire							
Self-rated depth of sleep (score)	35	4.5 ± 1.4	4.9 ± 1.4	29	4.9 ± 1.3	5.2 ± 1.6	0.57
Number of awakenings (times)	37	1.46 ± 1.26	1.49 ± 1.22	29	1.52 ± 1.24	1.41 ± 1.50	0.63
Sleep quality (score)	34	3.9 ± 1.1	4.2 ± 1.2	29	4.1 ± 1.0	4.3 ± 1.2	0.87
Alertness on waking (score)	34	3.4 ± 1.2	3.6 ± 1.4	28	3.4 ± 1.2	3.8 ± 1.1	0.64
Satisfaction with sleep (score)	34	3.2 ± 1.0	3.2 ± 1.1	29	3.3 ± 1.1	3.6 ± 0.9	0.30
Early morning awakening (n, % yes)	34	5, 14.7%	5, 14.7%	26	5, 19.2%	3, 11.5%	
Difficultly falling asleep (score)	35	1.2 ± 0.5	1.2 ± 0.5	29	1.3 ± 0.6	1.2 ± 0.5	0.23

Mean ± SD, a: night before forest walking, b: night after forest walking, c: repeated analysis of variance.

Regarding self-rated sleep by SMHSQ, no significant interaction was observed for any of the items. Since the main effects of time (the night before forest walking vs. the night after) were significant in self-rated depth of sleep (*p *= 0.03) and sleep quality (*p *= 0.04), these two items were improved regardless of timing of the session (forenoon or afternoon). The self-rated depth of sleep score increased after forest walking in both session groups (4.5 ± 1.4 to 4.9 ± 1.4 in the forenoon session; 4.9 ± 1.3 to 5.2 ± 1.6 in the afternoon session). The sleep quality score was also similarly increased regardless of the timing of the session (3.9 ± 1.1 to 4.2 ± 1.2 in the forenoon session; 4.1 ± 1.0 to 4.3 ± 1.2 in the afternoon session).

**Figure 1:**
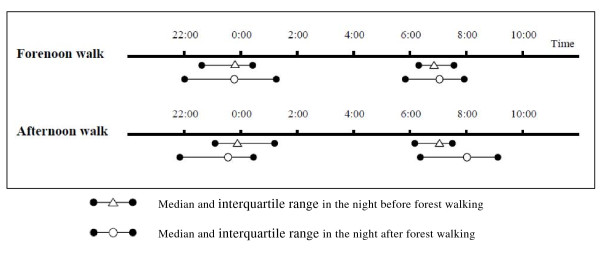
**Sleep start time (median and interquartile range) and sleep end time (median and interquartile range) the nights before and after forest walking for forenoon and afternoon walk subgroups**.

As presented in Figure 1, median sleep start time was less changed the night after forest walking compared with the night before for participants walking in the forenoon (23:48 to 23:46; n = 26) and participants walking in the afternoon (23:54 to 23:34, n = 16). Median sleep end time was delayed the night after forest walking compared with the night before for participants walking in the afternoon (7:04 to 8:01) whereas there was less extension for forenoon participants (6:52 to 7:04).

### Other relevant factors related to sleep improvement

Exploratory analysis revealed that a relevant factor related to sleep is decreased STAI-S scores (below or above average decrease) due to forest walking on sleep depth (interaction between decreased STAI-S scores and sleep depth, *p *= 0.002). In subgroups that had a greater than average decrease in STAI-S score after forest walking, the self-rated sleep depth score increased. By contrast, the score did not change in subgroups with a smaller than average decrease in STAI-S score after forest walking. Other factors were not relevant. Even if the model of repeated-measures ANOVA with both score change of STAI-S due to forest walking and forest walking session (forenoon and afternoon), the interaction between the score change of STAI-S by forest walking and sleep depth was also significant (*p *= 0.002).

## Discussion

This study suggests that 2 hours forest walking significantly lengthened mean actual sleep time and mean immobile time estimated by actigraphy and that sleep depth and sleep quality were improved, as evaluated by SMHSQ. In addition, some participants had a nap(s) after forest walking. Forest walking may contribute to improvement of subsequent sleep for individuals with sleep complaints. Exercise and emotional improvement initiated by walking in forested areas may bring both increased sleeping hours and improved subjective sleep quality.

Regarding objective sleep parameters, a previous review suggested that exercise increased total sleep time (TST) but did not significantly affect sleep onset latency (SOL) [39]; our results come close to supporting this. Recent studies reported that sleep emerges locally and is regulated in a use-dependent (homeostatic) manner [40,41]. The study showed that arm immobilization locally decreased slow wave activity in subsequent sleep; slow wave activity is thought to reflect sleep need [41]. Exercise during the prior wake period therefore might induce sleep.

Regarding emotional effects, this study suggests that 2 hours' forest walking significantly improved anxiety as measured by STAI-S. Furthermore, self-rated sleep depth for individuals whose STAI-S scores decreased by more than the average was much improved versus in individuals who had STAI-S scores decreased by less than the average--even when adjusted by forest walking session, which was a relevant factor for sleep improvement. From these results, it appears that improvement of self-rated sleep depth may depend on not only exercise but also improvement of psychological factors. Since forest walking, which does not require specific techniques, is a widely available activity, walking in forested areas may be a practical method to improve sleep that is easily applicable in daily life.

Our study suggests that afternoon forest walks had a greater effect on actual sleep time and immobile minutes than those taken in the forenoon. The time of day when exercise is undertaken is one factor related to the subsequent sleep response [23,39]. Youngstedt et al. [39] reported that SOL and wakefulness after sleep onset (WASO) were influenced by the time of day when exercise was completed, whereas TST was not influenced by the time of day. However, this study revealed that forest walking in the afternoon much improved actual sleep time and immobile minutes but not SOL compared with forest walking in the forenoon.

According to a two-process model [42,43] sleep and waking are regulated by circadian rhythms (Process C) and homeostasis (Process S). To examine why the afternoon session causes improvements in actual sleep time and immobile minutes, the core body temperature should be measured to determine the change of circadian phase (Process C) [42]. Since the measurement was not available in this study, we cannot conclude why the afternoon session caused improvements in actual sleep time and immobile minutes. However, one possible reason that the afternoon session brought improvements could be by homeostatic mechanism recovery after exercise (Process S). Exercise may amplify core body temperature. A steep decline of core body temperature before nocturnal sleep was reported to induce sleep [44]. The afternoon session, with a shorter interval between the end of walking and the onset of nocturnal sleep compared with the forenoon session, might be profitable for sleep improvements. On the other hand, because both forenoon and afternoon sessions are conducted in the daytime, they might not affect the circadian phase (Process C), as shown by the sleep start times of the forenoon and afternoon session participants which did not change on the nights before and after forest walking. Furthermore, the possibility still remains that the higher percentage of participants taking a nap(s) after forest walking in a forenoon session compared with an afternoon session was related to an apparent extension of nocturnal sleep duration in the participants in the afternoon sessions.

Intensity of exercise is also a factor related to subsequent sleep [23]. The intensity of forest walking in this study was considered light exercise on the Borg scale because the study was conducted in a forested area with few steep mountain paths. Since this study suggests that forest walking improved some sleep conditions, the intensity of exercise can be seen as appropriate.

This study has some limitations. First, there was no control group for walking in non-forested areas adjusted by exercise strength and light intensity. Therefore we cannot claim with certainty whether immediate improvements in the characteristics of sleep were brought on by walking only or by walking specifically carried out in forested areas. However, a previous study showed that STAI-S score was lower on a forest-walking day compared with another day with exercise in non-forested areas [22]. Furthermore, the present study suggests that improvement of anxiety measured by STAI-S was associated with self-rated sleep depth, even after adjusting for the timing of forest walking (forenoon vs. afternoon). From these two results, walking in forested areas, where emotional effects would be expected, might produce much improvement of sleep compared with walking in non-forested areas. Second, we evaluated only the immediately discernable effects of sleep.

The conclusions of this study are that 2 hours forest walking lengthened actual sleeping hours and immobile minutes and that it improved self-rated depth of sleep and sleep quality for individuals who had sleep complaints. The self-rated depth of sleep depended on emotional improvements. Forest walking in the afternoon prolonged actual sleeping hours and immobile minutes. Further studies using a randomized controlled trial design need to be carried out to evaluate whether a series of forest walks improves slight insomnia and slight sleep complaints. Such a study should reveal what factors in forest walking are responsible for improving sleep complaints.

## Competing interests

The authors declare that they have no competing interests.

## Authors' contributions

SM and TM deigned the study and collected the data. EM analyzed the data and MI provided advice on the data analysis. EM, MI, and MO interpreted the data. EM drafted the manuscript. MI, MO, and SM participated in revision of the manuscript. All authors have read and approved the final manuscript.

## Acknowledgements

The study was supported by the Ministry of Economy, Trade and Industry of Japan.
